# Supplementary material for: Modeling glioblastoma heterogeneity as a dynamic network of cell states
Source: Mol Syst Biol. 2021 Sep 16;17(9):e10105. doi: 10.15252/msb.202010105 (PMC8444284; doi:10.15252/msb.202010105)
Supplement: Supplementary file 5 — Source Data for Figure 3 [file MSB-17-e10105-s001.zip › Figure3A_sourcedata/GSEA_3065/nosigdb_state2.GseaPreranked.1622718419215/LARSSON_STATE_4.html]

Details for gene set LARSSON\_STATE\_4[GSEA]

|  || Dataset | state2 |
| Phenotype | NoPhenotypeAvailable |
| Upregulated in class | na\_pos |
| GeneSet | LARSSON\_STATE\_4 |
| Enrichment Score (ES) | 1.0000002 |
| Normalized Enrichment Score (NES) | 3.3396554 |
| Nominal p-value | 0.0 |
| FDR q-value | 0.0 |
| FWER p-Value | 0.0 |
Table: GSEA Results Summary

  

Fig 1: Enrichment plot: LARSSON\_STATE\_4      
 Profile of the Running ES Score & Positions of GeneSet Members on the Rank Ordered List

  

| PROBE | GENE SYMBOL | GENE\_TITLE | RANK IN GENE LIST | RANK METRIC SCORE | RUNNING ES | CORE ENRICHMENT || 1 | HIST1H4C |  |  | 0 | 1.309 | 0.0256 | Yes |
| 2 | HIST1H1B |  |  | 1 | 0.902 | 0.0432 | Yes |
| 3 | PCLAF |  |  | 2 | 0.852 | 0.0599 | Yes |
| 4 | HIST1H1D |  |  | 3 | 0.842 | 0.0764 | Yes |
| 5 | HMGB2 |  |  | 4 | 0.823 | 0.0925 | Yes |
| 6 | TYMS |  |  | 5 | 0.792 | 0.1079 | Yes |
| 7 | CENPU |  |  | 6 | 0.763 | 0.1229 | Yes |
| 8 | HIST1H1A |  |  | 7 | 0.760 | 0.1377 | Yes |
| 9 | H2AFZ |  |  | 8 | 0.735 | 0.1521 | Yes |
| 10 | UBE2C |  |  | 9 | 0.704 | 0.1659 | Yes |
| 11 | H2AFX |  |  | 10 | 0.675 | 0.1791 | Yes |
| 12 | DHFR |  |  | 11 | 0.644 | 0.1917 | Yes |
| 13 | UBE2T |  |  | 12 | 0.643 | 0.2042 | Yes |
| 14 | MCM7 |  |  | 13 | 0.626 | 0.2165 | Yes |
| 15 | MYBL2 |  |  | 14 | 0.600 | 0.2282 | Yes |
| 16 | TOP2A |  |  | 15 | 0.596 | 0.2399 | Yes |
| 17 | PCNA |  |  | 16 | 0.595 | 0.2515 | Yes |
| 18 | MKI67 |  |  | 17 | 0.590 | 0.2630 | Yes |
| 19 | CDK1 |  |  | 18 | 0.577 | 0.2743 | Yes |
| 20 | UBE2S |  |  | 19 | 0.574 | 0.2855 | Yes |
| 21 | MAD2L1 |  |  | 20 | 0.563 | 0.2965 | Yes |
| 22 | ATAD2 |  |  | 21 | 0.563 | 0.3076 | Yes |
| 23 | RRM2 |  |  | 22 | 0.556 | 0.3184 | Yes |
| 24 | CKS1B |  |  | 23 | 0.554 | 0.3293 | Yes |
| 25 | DUT |  |  | 24 | 0.552 | 0.3401 | Yes |
| 26 | HMGB1 |  |  | 25 | 0.549 | 0.3508 | Yes |
| 27 | NUDT1 |  |  | 26 | 0.541 | 0.3614 | Yes |
| 28 | CLSPN |  |  | 27 | 0.536 | 0.3719 | Yes |
| 29 | CKS2 |  |  | 28 | 0.532 | 0.3823 | Yes |
| 30 | TPX2 |  |  | 29 | 0.531 | 0.3927 | Yes |
| 31 | ESCO2 |  |  | 30 | 0.530 | 0.4030 | Yes |
| 32 | DEK |  |  | 31 | 0.526 | 0.4133 | Yes |
| 33 | FANCI |  |  | 32 | 0.521 | 0.4235 | Yes |
| 34 | STMN1 |  |  | 33 | 0.520 | 0.4337 | Yes |
| 35 | CENPF |  |  | 34 | 0.518 | 0.4438 | Yes |
| 36 | HMGN2 |  |  | 35 | 0.512 | 0.4538 | Yes |
| 37 | RPA3 |  |  | 36 | 0.511 | 0.4638 | Yes |
| 38 | GINS2 |  |  | 37 | 0.506 | 0.4737 | Yes |
| 39 | HIST1H1C |  |  | 38 | 0.498 | 0.4834 | Yes |
| 40 | SMC2 |  |  | 39 | 0.493 | 0.4931 | Yes |
| 41 | ORC6 |  |  | 40 | 0.493 | 0.5027 | Yes |
| 42 | DBF4 |  |  | 41 | 0.492 | 0.5123 | Yes |
| 43 | SMC4 |  |  | 42 | 0.489 | 0.5219 | Yes |
| 44 | TMPO |  |  | 43 | 0.489 | 0.5315 | Yes |
| 45 | CENPK |  |  | 44 | 0.488 | 0.5410 | Yes |
| 46 | ZWINT |  |  | 45 | 0.488 | 0.5505 | Yes |
| 47 | PBK |  |  | 46 | 0.484 | 0.5600 | Yes |
| 48 | NUSAP1 |  |  | 47 | 0.483 | 0.5694 | Yes |
| 49 | ANP32B |  |  | 48 | 0.474 | 0.5787 | Yes |
| 50 | PTMA |  |  | 49 | 0.474 | 0.5880 | Yes |
| 51 | KPNA2 |  |  | 50 | 0.472 | 0.5972 | Yes |
| 52 | MCM3 |  |  | 51 | 0.464 | 0.6063 | Yes |
| 53 | TUBA1B |  |  | 52 | 0.462 | 0.6153 | Yes |
| 54 | RFC2 |  |  | 53 | 0.460 | 0.6243 | Yes |
| 55 | CENPM |  |  | 54 | 0.460 | 0.6333 | Yes |
| 56 | CHAF1A |  |  | 55 | 0.457 | 0.6422 | Yes |
| 57 | TK1 |  |  | 56 | 0.456 | 0.6512 | Yes |
| 58 | UHRF1 |  |  | 57 | 0.454 | 0.6600 | Yes |
| 59 | SGO1 |  |  | 58 | 0.449 | 0.6688 | Yes |
| 60 | E2F1 |  |  | 59 | 0.446 | 0.6776 | Yes |
| 61 | NCAPG |  |  | 60 | 0.445 | 0.6863 | Yes |
| 62 | BIRC5 |  |  | 61 | 0.443 | 0.6949 | Yes |
| 63 | SIVA1 |  |  | 62 | 0.439 | 0.7035 | Yes |
| 64 | PTTG1 |  |  | 63 | 0.439 | 0.7121 | Yes |
| 65 | GMNN |  |  | 64 | 0.435 | 0.7206 | Yes |
| 66 | RANBP1 |  |  | 65 | 0.430 | 0.7290 | Yes |
| 67 | HIST1H1E |  |  | 66 | 0.429 | 0.7374 | Yes |
| 68 | FBXO5 |  |  | 67 | 0.420 | 0.7456 | Yes |
| 69 | CCNB1 |  |  | 68 | 0.420 | 0.7538 | Yes |
| 70 | LMNB1 |  |  | 69 | 0.417 | 0.7619 | Yes |
| 71 | EZH2 |  |  | 70 | 0.417 | 0.7701 | Yes |
| 72 | CENPH |  |  | 71 | 0.412 | 0.7782 | Yes |
| 73 | RRM1 |  |  | 72 | 0.412 | 0.7862 | Yes |
| 74 | LSM4 |  |  | 73 | 0.410 | 0.7942 | Yes |
| 75 | SNRPD1 |  |  | 74 | 0.408 | 0.8022 | Yes |
| 76 | LSM3 |  |  | 75 | 0.407 | 0.8102 | Yes |
| 77 | ASF1B |  |  | 76 | 0.407 | 0.8181 | Yes |
| 78 | SGO2 |  |  | 77 | 0.406 | 0.8261 | Yes |
| 79 | NUCKS1 |  |  | 78 | 0.404 | 0.8340 | Yes |
| 80 | MCM4 |  |  | 79 | 0.399 | 0.8418 | Yes |
| 81 | PIMREG |  |  | 80 | 0.398 | 0.8495 | Yes |
| 82 | TUBB |  |  | 81 | 0.397 | 0.8573 | Yes |
| 83 | H2AFV |  |  | 82 | 0.397 | 0.8651 | Yes |
| 84 | MCM5 |  |  | 83 | 0.394 | 0.8728 | Yes |
| 85 | KIF4A |  |  | 84 | 0.393 | 0.8805 | Yes |
| 86 | LSM5 |  |  | 85 | 0.393 | 0.8881 | Yes |
| 87 | KIF15 |  |  | 86 | 0.392 | 0.8958 | Yes |
| 88 | CENPN |  |  | 87 | 0.390 | 0.9034 | Yes |
| 89 | HNRNPD |  |  | 88 | 0.390 | 0.9111 | Yes |
| 90 | GTSE1 |  |  | 89 | 0.389 | 0.9187 | Yes |
| 91 | RAD51AP1 |  |  | 90 | 0.388 | 0.9262 | Yes |
| 92 | ALYREF |  |  | 91 | 0.382 | 0.9337 | Yes |
| 93 | BRCA1 |  |  | 92 | 0.381 | 0.9412 | Yes |
| 94 | PHF19 |  |  | 93 | 0.380 | 0.9486 | Yes |
| 95 | DTYMK |  |  | 94 | 0.379 | 0.9560 | Yes |
| 96 | CDC45 |  |  | 95 | 0.377 | 0.9634 | Yes |
| 97 | MND1 |  |  | 96 | 0.376 | 0.9707 | Yes |
| 98 | CDKN3 |  |  | 97 | 0.376 | 0.9781 | Yes |
| 99 | RAN |  |  | 98 | 0.375 | 0.9854 | Yes |
| 100 | TMSB15A |  |  | 99 | 0.374 | 0.9927 | Yes |
| 101 | MCM10 |  |  | 100 | 0.373 | 1.0000 | Yes |
Table: GSEA details [plain text format]

  

Fig 2: LARSSON\_STATE\_4: Random ES distribution      
 Gene set null distribution of ES for **LARSSON\_STATE\_4**
